# Optimising the unilateral DIEP flap breast reconstruction: A United Kingdom-Netherlands multi-centre comparative study

**DOI:** 10.1016/j.jpra.2026.01.014

**Published:** 2026-01-15

**Authors:** Maximilian Jacobi, Petko Shtarbanov, Punn Tannirandorn, Zahra Ahmed, Stephen Hamilton, Shadi Ghali, Afshin Mosahebi, Sophie Riesmeijer, Dariush Nikkhah, Hinne Rakhorst

**Affiliations:** a Department of Plastic, Reconstructive and Hand Surgery, Medisch Spectrum Twente (MST), Enschede, The Netherlands; b Department of Plastic, Reconstructive and Hand Surgery, Ziekenhuis Groep Twente (ZGT), Almelo, Hengelo, The Netherlands; c University College London, London, United Kingdom; d Department of Plastic and Reconstructive Surgery, Royal Free London NHS Foundation Trust, London, United Kingdom

**Keywords:** DIEP, Microsurgery, Breast, Reconstruction, Flaps, Complications (Clavien-Dindo)

## Abstract

**Background:**

The deep inferior epigastric perforator (DIEP) flap represents the gold standard in autologous breast reconstruction; however, it is restricted by prolonged operative duration and substantial demands on inpatient capacity. We compared postoperative outcomes across international centres with differing surgical models and perioperative pathways.

**Methods:**

A retrospective, two-centre cohort study was undertaken of unilateral DIEP flap reconstructions performed in the United Kingdom (UK) and the Netherlands (NL). The UK cohort (2018–2022) utilised a single-surgeon model with an enhanced recovery after surgery (ERAS) pathway, whereas the NL cohort (2013–2019) employed a co-surgeon model and lean optimisation without ERAS protocols. Patient demographics, operative characteristics, and postoperative complications were compared. Outcomes were analysed by complication severity (Clavien–Dindo), timing (<30 vs. ≥30 days), and potential predictors.

**Results:**

The cohort included 203 patients (203 flaps): 103 from the UK and 100 from NL. Immediate reconstruction predominated in the UK (70% vs. 1%, *P* < 0.001). Mean operative times were longer in the UK (464 vs. 330 min, *P* < 0.001), yet length of stay was shorter (4.8 vs. 5.9 days, *P* < 0.001). Overall complication rates were comparable (61% UK vs. 56% NL). Prolonged operative time predicted early minor complications in the UK cohort and was associated with late minor complications in UK-NL combined analyses. Increased BMI, chemotherapy, radiotherapy, and immediate reconstruction variably predicted fat necrosis and minor complications, while prior abdominal surgery was associated with late re-operation in NL. Flap loss rates (4–7%) were equivalent.

**Conclusions:**

The present study demonstrates that operative workflow, case-mix, and perioperative pathways may influence efficiency and complication profiles without altering flap survival. Pragmatic improvements include ERAS implementation, co-surgeon operating, lean optimisation, and tailored planning for higher-risk patients.

## Introduction

Breast reconstruction is an integral component of the clinical discussion for many patients following total mastectomy, with the potential to cause profound improvements in quality of life.^1^^,^^2^ Among available reconstructive options, autologous free flaps have shown superior patient satisfaction compared to implant-based options and avoid risks associated with implants, such as capsular contracture.^3^, ^4^, ^5^, ^6^ The deep inferior epigastric perforator (DIEP) flap has emerged as the gold standard approach for autologous breast reconstruction.^7^ The DIEP flap offers faster recovery, decreased postoperative pain, and reduced donor-site morbidity compared to non-muscle sparing alternatives. Growing awareness of these benefits has contributed to rising global demand.

Nevertheless, the DIEP flap reconstruction is resource-intensive, with operative times that often extend to 8–12 hours^8^^,^^9^ and non-trivial demands on perioperative care and inpatient capacity. Prolonged operative time has been associated with increased postoperative complications across various surgical disciplines,^10^^,^^11^ and in DIEP flap cohorts reported predominantly from single-centre studies.^12^^,^^13^ In parallel, constrained operating room capacity has contributed to longer waiting lists in many centres. Strategies to improve efficiency, such as lean process optimisation adapted from the Toyota production system,^14^, ^15^, ^16^ structured team configurations including co-surgeon operating, and enhanced recovery after surgery (ERAS) pathways, have been associated with shorter operative times and strengthened perioperative metrics in selected settings, while recognising that effects vary by context.^17^

To our knowledge, few, if any, studies have examined these issues using an international, multi-centre comparative cohort focused specifically on the DIEP flap breast reconstruction. Multiple patient- and operation-related factors may influence outcomes and efficiency; a careful assessment can facilitate like-for-like comparisons between centres and highlight practice areas that merit further evaluation.

In this retrospective two-centre study of unilateral, DIEP flap-only breast reconstructions, including one cohort from the Netherlands (NL) and one from the United Kingdom (UK), we aimed to compare case-mix, operative characteristics, and postoperative outcomes, and explore associations with early and late complications, including events requiring surgical or radiological intervention. The objective was to generate pragmatic, hypothesis-forming insights to inform service planning and surgical efficiency while acknowledging the limits of retrospective analysis.

## Patients and methods

A retrospective review was carried out, according to STROBE guidelines, of patients treated with unilateral DIEP flaps performed at two separate centres – one site in NL and another in the UK. The NL cohort covered reconstructions performed between 2013 and 2019 using the co-surgeon model (with two different senior microsurgeons leading the operation concurrently), and the UK cohort ranged from 2018 to 2022 with cases led by eleven different senior microsurgeons (with the single senior surgeon model). Moreover, in the NL cohort, lean optimisation was deliberately implemented and iteratively refined (in continuous‐improvement cycle) as part of establishing the DIEP flap pathway. In brief, the lean pathway comprised parallelisation of flap harvest and recipient-site preparation, a standardised microsurgical workflow (including routine use of venous couplers and a “redo-if-any-doubt” rule), predefined decision algorithms for internal mammary exposure and rib management, and task-shifting of non-critical closure steps to junior staff. The practical examples of these workflow optimisation models are further described in Supplement 1. For context, some of the microsurgeons regularly practiced across different sites while the present study analysed data from a single site only in each country, and therefore per-surgeon case counts derived from these data must not be interpreted as total annual workload or overall experience.

Each centre employed their own standardised protocols. Prospectively-kept medical records were used to obtain anonymised datasets. Eligible cases included elective unilateral DIEP flap breast reconstructions, categorised as immediate (performed concurrently with mastectomy) or delayed (performed after a separate mastectomy).

Indications for reconstruction included post-mastectomy for primary breast cancer or risk-reduction. Patients were excluded due to missing key variables, bilateral operations, bipedicled arterial anastomoses, non-DIEP flap reconstructions, or a postoperative follow-up of less than 6 months. Postoperative complication types and other retrospective data collected are detailed in Supplement 2.

Operative time was defined as skin incision to closure time inclusive of the duration of the mastectomy. Incidence of all adverse outcomes and flap-site complications were calculated per patient. Postoperative follow-up care was consistent across patients and performed by trained personnel. The UK site followed a standardised ERAS protocol throughout the study period, and consistently applied it to all patients (see Supplement 3). We categorised complications as early (<30 days) or late (≥30 days) depending on postoperative day of occurrence, and their severity was classified according to the Clavien-Dindo scale,^18^ with grade 2 or grade ≥3 being the focus of interest.

### Statistical analysis

Cohorts of each centre were assessed separately and pooled where applicable. Descriptive statistics included mean and standard deviation and counts (*n*) and percentage for nominal data. Differences in continuous variables were assessed with independent-samples t-test. Logistic regression was limited to univariable models to examine the effect of continuous predictors and obtain odds ratios (OR) and 95% confidence intervals (CI); binary predictors were analysed with chi-square tests.

In a subsequent step, multivariable logistic regression analyses were performed for all outcomes where at least one predictor reached statistical significance in univariable testing, whether in the UK, NL, or pooled cohort. This approach served to evaluate whether the predictors identified in univariable analyses remained independently associated with the outcome when controlling for potential confounders. All multivariable models included the following covariables assessed in Table 3, Table 4, Table 5, Table 6.

To aid clinical interpretation, the association of operative time with outcomes is presented per minute and additionally per 60-min increase. Analyses used a complete-case approach; denominators were reported per analysis. Given the exploratory nature of the study, no adjustments for multiple comparisons were applied, and results should be interpreted as hypothesis-generating. A two-tailed *P* < 0.05 was considered statistically significant. All analyses were performed using SPSS 29.0 (IBM, Armonk, NY, USA) software.

## Results

Our final cohort included a total of 203 patients (203 flaps) undergoing unilateral DIEP flap breast reconstruction, with 103 cases from the UK and 100 from NL. Cohort baseline characteristics are described in Table 1. Average age, body mass index (BMI), and smoking rates were comparable across centres, while NL patients had more comorbidities. Significant between-centre differences included higher prior abdominal surgery in the UK (41% vs. 9%, *P* < 0.001) and higher rates of chemotherapy (48% vs. 33%, *P* = 0.030) and radiotherapy (45% vs. 30%, *P* = 0.028) in NL. Immediate reconstruction was predominant in the UK (70% vs. 1%, *P* < 0.001). Mean operative time was longer in the UK (464 ± 74 min) than in NL (330 ± 81 min, *P* < 0.001), and postoperative length of stay was shorter in the UK (4.8 ± 1.0 vs. 5.9 ± 1.8 days, *P* < 0.001).

**Table 1:** Baseline characteristics.

Variable assessed	Unit UK	Unit NL	*P*
Total patients, *n*	103	100	
Mean age in years ± SD (range)	51.1 ± 7.7 (32–70)	51.8 ± 8.1 (30–77)	0.504
Mean BMI in kgm^-2^ ± SD (range)	28.1 ± 3.9 (17–40)	27.1 ± 3.0 (20–35)	0.037*
Mean operative time in min ± SD (range)	464 ± 74 (315–729)	330 ± 81 (177–613)	*P*<0.001*
Mean length of hospital stay in days ± SD (range)	4.8 ± 1.0 (3–8)	5.9 ± 1.8 (3–15)	*P*<0.001*
Positive smoking status, *n* (%)	9 (8.7%)	6 (6.0%)	0.458
Hypertension and diabetes mellitus, *n* (%)	11 (10.7%)	17 (17.0%)	0.267
Prior abdominal surgery, *n* (%)	42 (40.8%)	9 (9.0%)	*P*<0.001*
Chemotherapy, *n* (%)	34 (33.0%)	49 (49.0%)	0.030*
Past radiotherapy, *n* (%)	31 (30.1%)	45 (45.0%)	0.028*
Immediate procedure, *n* (%)	72 (69.9%)	1 (1.0%)	*P*<0.001*

Abbreviations: BMI, body mass index; NL, The Netherlands; SD, standard deviation; UK, United Kingdom.

Significantly more immediate reconstructions occurred in the UK unit, with a significantly longer total operative time and a shorter length of postoperative stay relative to the NL unit. Demographically, the UK unit had patients with higher BMI and higher frequency of prior abdominal surgery, but lower proportions of patients with previous or ongoing cancer therapy relative to the NL unit.

⁎ In bold: *P* < 0.05 (significant).

The overall proportion of patients with at least one complication did not differ significantly between centres (61% UK vs. 56% NL, *P* = 0.450) as summarised in Table 2. For major complications requiring intervention (Clavien–Dindo grade 3 or higher), prior abdominal surgery was the only significant predictor (Table 3). This association was observed in the NL cohort, where prior abdominal surgery predicted late re-operations (*P* = 0.007) and the findings were confirmed in multivariable analysis (OR: 8.20, 95% CI: 1.27–52.84, *P* = 0.027).

**Table 2:** Overview of complications in the entire cohorts.

Complication type incidence	Unit UK		Unit NL	
	Early	Late	Early	Late
Total number of complications, *n* (%)	90 (100%)	34 (100%)	89 (100%)	23 (100%)
Fat necrosis, *n* (%)	9 (10.0%)	18 (52.9%)	17 (19.1%)	8 (34.8%)
Flap loss, *n* (%)	4 (4.4%)	0 (0.0%)	6 (6.7%)	0 (0.0%)
Skin necrosis abdomen, *n* (%)	4 (4.4%)	1 (2.9%)	3 (3.4%)	1 (4.3%)
Skin necrosis breast, *n* (%)	11 (12.2%)	3 (8.8%)	1 (1.1%)	0 (0.0%)
Wound dehiscence, *n* (%)	19 (21.1%)	9 (26.5%)	17 (19.1%)	4 (17.4%)
Haematoma, *n* (%)	7 (7.8%)	0 (0.0%)	7 (7.9%)	0 (0.0%)
Infection, *n* (%)	9 (10.0%)	0 (0.0%)	12 (13.5%)	1 (4.3%)
Seroma, *n* (%)	15 (16.7%)	3 (8.8%)	4 (4.5%)	1 (4.3%)
Any re-operation, *n* (%)	12 (13.3%)	0 (0.0%)	22 (24.7%)	8 (34.8%)

Abbreviations: NL, The Netherlands; UK, United Kingdom.

Early, within 30 days postoperatively; Late, after 30 days postoperatively.

**Table 3:** Identifying predictors for postoperative re-operation rate after 30 days (any Clavien-Dindo type ≥3).

Variable assessed	Unit UK	Unit NL
*Logistic regression* Unadjusted OR (95% CI), *P*		
Operative time, min	N/A	1.001 (0.99–1.01), *P* = 0.892
Age, years	N/A	1.03 (0.95–1.13), *P* = 0.478
BMI, kgm^-2^	N/A	1.08 (0.85–1.38), *P* = 0.512
*Chi-square* *n* in group affected by complication/total in group (%), *P*		
Positive smoking status	N/A	1/6 (16.7%), *P* = 0.420
Hypertension and diabetes mellitus	N/A	2/17 (11.8%), *P* = 0.469
Prior abdominal surgery	N/A	3/9 (33.3%), *P*=0.007*
Past radiotherapy	N/A	3/45 (6.7%), *P* = 0.657
Chemotherapy	N/A	3/49 (6.1%), *P* = 0.535
Immediate procedure	N/A	1/1 (100%)
Axillary lymph node clearance	N/A	N/A
Any additional procedures	N/A	N/A

Abbreviations: BMI, body mass index; CI, confidence interval; N/A, not available (no complications of the given type occurred in this group); NL, The Netherlands; OR, odds ratio; UK, United Kingdom.

Prior abdominal surgery was the only statistically significant predictor of complications that required an intervention (Clavien-Dindo ≥3). However, it only reached significance in the analysis of the Dutch cohort.

⁎ In bold: *P* < 0.05 (significant).

For prespecified target complications, early fat necrosis within 30 days was associated with higher BMI in the UK cohort (OR: 1.22, 95% CI: 1.02–1.47, *P* = 0.029; multivariable OR: 1.28, 95% CI: 1.02–1.60, *P* = 0.035) and in pooled analysis (OR: 1.14, 95% CI: 1.01–1.29, *P* = 0.041), albeit without confirmation in the multivariabe model (OR: 1.12, 95% CI: 0.98–1.27, *P* = 0.088), and with prior abdominal surgery in NL (*P* = 0.041) again in univariable only (multivariable OR: 3.71, 95% CI: 0.82–16.70, *P* = 0.088) as shown in Table 4. Beyond 30 days, fat necrosis was associated with prior radiotherapy in pooled univariable analysis (*P* = 0.040; multivariable OR: 0.44, 95% CI: 0.14–1.40, *P* = 0.165) and with immediate reconstruction in pooled univariable analysis (*P* = 0.013, multivariable OR: 1.88, 95% CI: 0.65–5.40, *P* = 0.243) as shown in Table 4. No significant predictors were identified for flap loss (Supplement 4). For breast-skin necrosis, 12 of 15 cases occurred within 30 days and 14 followed immediate reconstruction (*P* < 0.001); no other significant predictors were observed.

**Table 4:** Identifying predictors for postoperative fat necrosis within and after 30 days.

Variable assessed	Fat necrosis within 30 days			Fat necrosis after 30 days		
	Unit UK	Unit NL	Pooled	Unit UK	Unit NL	Pooled
*Logistic regression* Unadjusted OR (95% CI), *P*						
Operative time, min	1.003 (0.99–1.01), *P* = 0.528	0.998 (0.99–1.01), *P* = 0.534	1.00 (0.99–1.01), *P* = 0.881	0.999 (0.99–1.01), *P* = 0.765	1.002 (0.99–1.01), *P* = 0.633	1.00 (0.10–1.01), *P* = 0.956
Age, years	1.001 (0.92–1.09), *P* = 0.986	1.04 (0.97–1.11), *P* = 0.247	1.03 (0.97–1.08), *P* = 0.342	0.99 (0.92–1.06), *P* = 0.705	1.04 (0.95–1.14), *P* = 0.398	1.01 (0.95–1.060), *P* = 0.843
BMI, kgm^-2^	1.22 (1.02–1.47), *P*=0.029*	1.06 (0.89–1.26), *P* = 0.500	1.14 (1.01–1.29), *P*=0.041*	0.97 (0.85–1.12), *P* = 0.641	0.88 (0.69–1.12), *P* = 0.293	0.95 (0.84–1.07), *P* = 0.359
*Chi-square* *n* in group affected by complication/total in group (%), *P*						
Positive smoking status	1/9 (11.1%), *P* = 0.792	1/6 (16.7%), *P* = 0.982	2/15 (13.3%), *P* = 0.950	1/9 (11.1%), *P* = 0.599	1/6 (16.7%), *P* = 0.420	2/15 (13.3%), *P* = 0.950
Hypertension and diabetes mellitus	1/11 (9.1%), *P* = 0.965	2/17 (11.8%), *P* = 0.601	3/28 (10.7%), *P* = 0.777	1/11 (9.1%), *P* = 0.438	1/17 (5.9%), *P* = 0.778	2/28 (7.1%), *P* = 0.367
Prior abdominal surgery	4/42 (9.5%), *P* = 0.815	4/9 (44.4%), *P*=0.041*	8/53 (15.1%), *P* = 0.519	7/42 (16.7%), *P* = 0.858	2/9 (22.2%), *P* = 0.140	9/51 (17.7%), *P* = 0.260
Past radiotherapy	4/31 (12.9%), *P* = 0.326	10/45 (22.2%), *P* = 0.209	14/76 (18.4%), *P* = 0.064	3/31 (9.7%), *P* = 0.171	2/45 (4.4%), *P* = 0.236	5/76 (6.6%), *P*=0.040*
Chemotherapy	4/34 (11.8%), *P* = 0.445	8/49 (16.7%), *P* = 0.932	12/83 (14.5%), *P* = 0.522	4/34 (11.8%), *P* = 0.284	2/49 (4.1%), *P* = 0.175	6/83 (7.2%), *P* = 0.054
Immediate procedure	6/72 (8.3%), *P* = 0.825	N/A	N/A	15/72 (20.8%), *P* = 0.171	1/1 (100%)	16/73 (21.9%), *P*=0.013*
Axillary lymph node clearance	2/15 (13.3%), *P* = 0.495	N/A	N/A	3/15 (20.0%), *P* = 0.781	N/A	N/A
Any additional procedures	N/A	1/16 (6.3%), *P* = 0.212	N/A	N/A	N/A	N/A

Abbreviations: BMI, body mass index; CI, confidence interval; N/A, not available (no complications of the given type occurred in this group); NL, The Netherlands; OR, odds ratio; UK, United Kingdom.

For fat necrosis within 30 days, prior abdominal surgery was a significant predictor in the Dutch cohort. Increasing BMI significantly predicted fat necrosis within 30 days in the British cohort and the pooled analysis. Past radiotherapy and immediate procedures both were significant predictors of fat necrosis after 30 days in the pooled analysis only.

⁎ In bold: *P* < 0.05 (significant).

For early minor complications (Clavien–Dindo grade 2 or less within 30 days), operative time significantly predicted events in the UK cohort (OR: 1.009 per minute, 95% CI: 1.002–1.015, *P* = 0.006; equivalent to OR: 1.71 per 60 min, 95% CI: 1.13–2.44; multivariable per 60 min OR: 1.25, 95% CI: 1.01–1.54, *P* = 0.041), as well as immediate procedure in multivariable models only (OR: 3.44, 95% CI: 1.06–11.13, *P* = 0.040). Increasing BMI was a significant predictor in pooled data (OR: 1.14, 95% CI: 1.04–1.24, *P* = 0.003; multivariable OR: 1.13, 95% CI: 1.03–1.23, *P* = 0.012). Chemotherapy was also significant for early complications in the UK and in pooled analysis (both *P* = 0.003; UK multivariable OR: 3.14, 95% CI: 1.05–9.41, *P* = 0.041 and pooled OR: 2.63, 95% CI: 1.34–5.14, *P* = 0.005). In the NL cohort, performing any additional procedure was associated with increased odds of early minor complications in univariable analysis (*P* = 0.006) and remained significant after adjustment (adjusted OR: 11.0, 95% CI 2.23–55.60, *P* = 0.003) (Table 5).

**Table 5:** Identifying predictors for postoperative complications within and after 30 days (any Clavien-Dindo type).

Variable assessed	Any complication within 30 days			Any complication after 30 days		
	Unit UK	Unit NL	Pooled	Unit UK	Unit NL	Pooled
*Logistic regression* Unadjusted OR (95% CI), *P*						
Operative time, min	1.009 (1.002–1.015), *P*=0.006*	1.002 (0.997–1.006), *P* = 0.547	1.002 (0.999–1.005), *P* = 0.137	1.003 (0.997–1.009), *P* = 0.301	1.003 (0.997–1.010), *P* = 0.282	1.005 (1.001–1.008), *P*=0.005*
Age, years	0.95 (0.90–1.001), *P* = 0.057	1.02 (0.97–1.07), *P* = 0.407	0.99 (0.95–1.02), *P* = 0.471	0.98 (0.93–1.05), *P* = 0.506	1.01 (0.95–1.08), *P* = 0.708	0.99 (0.95–1.03), *P* = 0.683
BMI, kgm^-2^	1.11 (0.99–1.23), *P* = 0.064	1.21 (1.05–1.40), *P* = 0.010	1.14 (1.04–1.24), *P*=0.003*	0.964 (0.860–1.080), *P* = 0.526	0.895 (0.745–1.074), *P* = 0.232	0.97 (0.88–1.06), *P* = 0.472
*Chi-square* *n* in group affected by complication/total in group (%), *P*						
Positive smoking status	5/9 (55.6%), *P* = 0.573	4/6 (66.7%), *P* = 0.400	9/15 (60.0%), *P* = 0.345	3/9 (33.3%), *P* = 0.948	2/6 (33.3%), *P* = 0.232	5/15 (33.3%), *P* = 0.394
Hypertension and diabetes mellitus	4/11 (36.4%), *P* = 0.231	8/16 (50.0%), *P* = 1.000	12/28 (42.9%), *P* = 0.416	3/11 (27.3%), *P* = 0.703	2/17 (11.8%), *P* = 0.677	5/28 (17.9%), *P* = 0.455
Prior abdominal surgery	21/42 (50.0%), *P* = 0.566	7/9 (77.8%), *P* = 0.182	28/51 (54.9%), *P* = 0.351	13/42 (31.0%), *P* = 0.800	4/9 (44.4%), *P*=0.029*	17/51 (33.3%), *P* = 0.100
Past radiotherapy	16/31 (51.6%), *P* = 0.504	26/45 (57.8%), *P* = 0.159	42/76 (55.3%), *P* = 0.123	5/31 (16.1%), *P*=0.021*	5/45 (11.1%), *P* = 0.228	10/76 (13.2%), *P*=0.004*
Chemotherapy	23/34 (67.7%), *P*=0.003*	27/49 (55.1%), *P* = 0.230	50/83 (60.2%), *P*=0.003*	7/34 (20.6%), *P* = 0.073	5/48 (10.4%), *P* = 0.143	12/83 (14.5%), *P*=0.008*
Immediate procedure	36/72 (50.0%), *P* = 0.292	1/1 (100%)	37/73 (50.7%), *P* = 0.607	27/72 (37.5%), *P* = 0.064	1/1 (100%)	28/73 (38.4%), *P*=0.001*
Axillary lymph node clearance	9/15 (60.0%), *P* = 0.260	N/A	N/A	7/15 (46.7%), *P* = 0.129	N/A	N/A
Any additional procedures	N/A	3/16 (18.8%), *P*=0.006*	N/A	N/A	N/A	N/A

Abbreviations: BMI, body mass index; CI, confidence interval; N/A, not available (no complications of the given type occurred in this group); NL, The Netherlands; OR, odds ratio; UK, United Kingdom.

With regards to any complication within 30 days, any additional procedures emerged as a predictor in the Dutch cohort and operative time as a predictor in the British cohort. Increasing BMI emerged as a significant risk factor in the pooled analysis, and chemotherapy was significant in both the pooled and British cohort. In terms of predicting any complication after 30 days, operative time, chemotherapy and immediate procedures all reached significance in the pooled analyses only, while prior abdominal surgery reached significance only in the Dutch cohort. Past radiotherapy stood out in reaching significance in both the British cohort and pooled analysis for complications after 30 days.

⁎ In bold: *P* < 0.05 (significant).

For late minor complications (30 days or later), pooled analyses showed significant associations with operative time in univariable only (OR: 1.005 per minute, 95% CI: 1.001–1.008, *P* = 0.005; equivalent to OR: 1.35 per 60 min, 95% CI: 1.06–1.61; multivariable OR: 1.09, 95% CI: 0.96–1.23, *P* = 0.176), chemotherapy (*P* = 0.008; multivariate OR: 0.55, 95% CI: 0.24–1.25), *P* = 0.152), immediate reconstruction (*P* = 0.001; multivariable OR: 1.65, 95% CI: 0.72–3.79, *P* = 0.236), and axillary lymph-node clearance (*P* = 0.020). Prior radiotherapy predicted late minor complications in pooled univariable analysis (*P* = 0.004; multivariable OR: 0.45, 95% CI: 0.19–1.09, *P* = 0.076) and in the UK cohort (*P* = 0.021; multivariable OR: 0.46, 95% CI: 0.13–1.71, *P* = 0.247). Prior abdominal surgery was significant only in NL (*P* = 0.029; multivariable OR: 8.95, 95% CI: 1.54–51.99, *P* = 0.015) as shown in Table 5.

In the delayed-only subgroup, mean operative time remained longer in the UK than in NL (457 ± 91 vs. 328 ± 80 min, *P* < 0.001). The proportion of patients with at least one complication did not differ significantly between centres in delayed cases (48% UK vs. 56% NL, *P* = 0.480). Predictors of complications in delayed reconstructions are detailed in Table 6. Only BMI (adjusted OR: 1.16, 95% CI: 1.002–1.35, *P* = 0.046) and prior abdominal surgery (adjusted OR: 8.81, 95% CI: 1.01–76.64, *P* = 0.049) turned out to be significant in multivariable models of the NL cohort.

**Table 6:** Identifying predictors for any complications occurring in delayed reconstructions only (combined within and after 30 days; any Clavien-Dindo type).

Variable assessed	Unit UK (*n* = 31)	Unit NL (*n* = 99)
*Logistic regression* Unadjusted OR (95% CI), *P*		
Operative time, min	1.014 (1.002–1.025), *P*=0.017*	1.001 (0.996–1.006), *P* = 0.599
Age, years	0.89 (0.81–0.99), *P*=0.031*	1.02 (0.97–1.07). *P* = 0.457
BMI, kgm^-2^	1.09 (0.93–1.29), *P* = 0.297	1.12 (1.01–1.34), *P*=0.034*
*Chi-square* *n* in group affected by complication/total in group (%), *P*		
Positive smoking status	0/2 (0.0%), *P* = 0.131	1/6 (16.7%), *P* = 0.158
Hypertension and diabetes mellitus	3/4 (75.0%), *P* = 0.254	7/16 (43.8%), *P* = 0.452
Prior abdominal surgery	6/12 (50.0%), *P* = 0.886	1/9 (11.1%), *P*=0.021*
Past radiotherapy	7/16 (43.8%), *P* = 0.594	28/45 (62.2%), *P* = 0.223
Chemotherapy	8/15 (53.3%), *P* = 0.594	27/48 (56.3%), *P* = 0.719
Axillary lymph node clearance	N/A	N/A
Any additional procedures	N/A	**3/16 (18.8%),*****P***= 0.001*

Abbreviations: BMI, body mass index; CI, confidence interval; N/A, not available (no complications of the given type occurred in this group); NL, The Netherlands; OR, odds ratio; UK, United Kingdom.

In the UK cohort, longer operative time and younger age were linked to higher complication risk, while these associations were absent in the Dutch cohort. Body mass index predicted complications only in the Dutch group. Smoking, hypertension, diabetes, radiotherapy, and chemotherapy were not significant predictors in either cohort. Prior abdominal surgery increased complication risk in the Dutch cohort but not in the UK group.

⁎ In bold: *P* < 0.05 (significant).

## Discussion

This study identified significant differences between the United Kingdom and the Netherlands cohorts undergoing unilateral DIEP flap breast reconstruction, specifically regarding patient characteristics, surgical practices, operative duration, and postoperative outcomes. Clinically notable findings include longer operative times (including in delayed-only cases), but shorter hospital stays in the UK in the context of an ERAS protocol in place throughout the study period, with higher prevalence of immediate reconstructions in the UK versus delayed reconstructions in the Netherlands, increased rates of prior abdominal surgery in the UK cohort, and differing complication profiles, such as increased seromas and breast-skin necrosis in the UK, and more early fat necrosis in the Netherlands cohort. These key results and their implications for practice are discussed below.

### Operative time

Longer operative time in this cohort was associated with selected complications after adjustment for timing windows, with effect sizes that appear small per minute but become clinically meaningful when expressed per hour. In pooled analyses, late minor complications increased with operative time (OR: 1.005 per minute; OR: 1.35 per 60 min), and in the UK cohort, early minor complications were likewise associated with duration (OR: 1.009 per minute; OR: 1.71 per 60 min). These observations were directionally consistent with reports linking prolonged procedures to higher postoperative morbidity across surgical disciplines and in autologous breast reconstruction specifically.^10^^,^^12^^,^^13^ We emphasise that these associations are not causal, and likely reflect a composite of workflow, team configuration, case complexity, and training exposure for surgical residents.

Differences in operative workflow likely made the dominant contributions to the between-centre gap in duration. The Netherlands site used a co-surgeon model and formal lean pathways,^17^^,^^19^ whereas the UK site used a single-surgeon model. We also note that surgeon activity in both countries spanned multiple institutions; our dataset captured one centre per country and DIEP flap-only activity, and hence per-surgeon counts here must not be interpreted as overall experience. Accordingly, we discuss experience density at the included site, co-surgeon working, case-mix, and process optimisation as plausible contributors to the observed time differences.

### Immediate versus delayed operations

A substantially higher proportion of immediate reconstructions in the UK cohort potentially contributed to the observed differences in operative time, and we acknowledge the limited power of the delayed-only subgroup. Although flap harvest was initiated concurrently with the mastectomy and axillary procedures, immediate reconstructions inherently required additional steps, which extended the total duration. This pattern reflects broader international practice: in the UK most patients undergo immediate reconstruction following mastectomy,^20^ whereas delayed reconstruction remains more common in many centres in the Netherlands.^21^

In our cohort, the absence of a statistically significant increase in major complications with immediate reconstruction is clinically relevant and aligns with reports that immediate procedures can be performed safely in well-selected patients.^22^ We observed mastectomy-skin flap necrosis clustered as an early event and occurred predominantly after immediate reconstruction. Importantly, we do not ascribe a causal mechanism to operative duration. Differences in mastectomy technique, for example skin-sparing versus non-skin-sparing, may influence necrosis risk and could not be fully characterised in this retrospective dataset.

Delayed reconstructions, although often requiring multiple stages, may allow more deliberate operative planning and have been associated in some studies with lower overall and major complication rates while achieving comparable health-related quality of life outcomes.^23^^,^^24^ Much of the patient-reported outcome literature combines implant-based and autologous reconstructions, which complicates direct comparisons to a DIEP flap-only cohort. Where such studies are cited, they are used to contextualise counselling rather than to infer implant-specific complication patterns to autologous flaps. In the Netherlands, national guidance stipulating a maximum of 6 weeks between diagnosis and mastectomy, with or without reconstruction, further shapes timing and case selection.^25^ Delayed reconstruction also remains an important option for patients with relevant comorbidities or where the extent of surgery increases perioperative risk, while immediate reconstruction continues to be favoured by many patients and surgeons because of its positive psychosocial, sexual, and emotional impact.^26^

Looking ahead, operative times may increase as centres, including the UK site in this study, adopt one-stop strategies that incorporate contralateral symmetrisation during the index reconstruction.^27^

### Flap loss and vascular complications

We found comparable flap success rates (94–96%) across both centres despite differences in institutional settings (academic versus specialist), confirming the reliability of DIEP flap procedures. In broader practice, many centres have adopted adjunctive techniques to further improve outcomes, including intraoperative assessment of flap perfusion and anastomotic patency using indocyanine green fluorescence angiography,^28^ as well as establishing dual venous outflow by anastomosing a second flap vein to a recipient vein to reduce the risk of venous congestion.^29^

### Patient selection

In our cohort, prior abdominal surgery was more common in the UK. Within-centre analyses showed that prior abdominal surgery was associated with selected adverse outcomes in the Netherlands cohort, including early fat necrosis and late re-operation, while these associations were not retained in pooled analyses. We therefore interpret prior abdominal surgery as a planning consideration rather than a general contraindication. The dataset recorded prior abdominal surgery as a binary variable and did not capture scar pattern or location, which can influence perforator selection and wound healing. The cohort comprised true DIEP flaps only; any intra-operative conversions to other reconstructive types were excluded. These observations are consistent with reports that prior abdominal operations can disrupt lymphatics and compromise vascularity, contributing to seroma, fat necrosis, and delayed donor-site healing.^30^, ^31^, ^32^, ^33^

Radiotherapy requires careful distinction between flaps transferred into previously irradiated fields and flaps that receive radiotherapy after transfer. In this study, “radiotherapy” reflected a history of chest-wall irradiation prior to flap transfer; consistent data on postoperative radiotherapy to the flap were not available. Against that definition, we observed associations between prior radiotherapy and late complications, including fat necrosis, in pooled and centre-specific analyses, without a signal for increased major complications or flap loss. Autologous reconstruction is generally considered more tolerant of radiation than implant-based reconstruction, although tissue quality and vascularity in irradiated fields still warrant tailored planning.^34^^,^^35^ Radiation-related microvascular and stromal changes have been linked to higher risks of fat necrosis and wound problems, particularly in low-turnover adipose tissues.^36^^,^^37^ Recent reports suggested that autologous reconstruction performed within a relatively short interval after completing radiotherapy, approximately 3 months, can be feasible and safe in selected patients, while longer-term outcomes and emerging strategies such as neoadjuvant radiation before mastectomy require further study.^38^^,^^39^

Finally, chemotherapy was associated with early and late minor complications in pooled and centre-specific analyses. As with radiotherapy and prior abdominal surgery, we interpret these as associations that may reflect case-mix and treatment timing rather than direct causation, underscoring the importance of multidisciplinary selection and optimisation before DIEP flap reconstruction.

### Length of postoperative hospital stay

Postoperative hospital stays differed between the two centres, with longer inpatient stays observed in the Netherlands cohort. During the study period, the UK centre implemented an ERAS protocol (Supplement 3), which has been shown in previous studies to significantly reduce hospital length of stay following autologous breast reconstruction.^40^ In contrast, the Netherlands centre had not formally adopted ERAS protocols at the time, likely contributing to prolonged admissions. To note, differences in length of stay could not be attributed to ERAS alone. Broader experience describes postoperative stays for autologous breast reconstruction typically ranging between one and 7 days, influenced by patient comfort, institutional policies, and confidence in recovery versus fear of complications. Delays in patient discharge over weekends due to reduced staffing, a challenge faced by both centres, may have further extended hospital stays. To optimise recovery pathways, emphasis should be placed on pragmatic targets to support safe earlier discharge including structured preoperative education for patients and staff, standardised criteria for mobilisation and drain management, and routine reporting of length of stay alongside complication rates as complementary quality indicators. Importantly, we did not observe evidence that the shorter postoperative stay in the UK compromised detection of complications. All patients were reviewed in a structured outpatient dressing clinic following discharge, and unplanned readmissions and medical complications, including venous thromboembolism, were recorded (Supplement 2). In keeping with previous DIEP flap series, symptomatic deep vein thrombosis and pulmonary embolism were uncommon and did not cluster in the cohort with earlier discharge. Length of stay was therefore not modelled as an independent predictor in our regression analyses because it is strongly collinear with centre, ERAS implementation, and case-mix, limiting causal interpretation.

### Limitations

This retrospective, two-centre study was subject to selection bias and cannot support causal inference. Important unmeasured confounders included differences in pathways and case-mix between centres, such as the high rate of immediate reconstruction in the UK, the co-surgeon and lean processes in the Netherlands, trainee participation, and operative complexity. Additionally, variations in operators’ microsurgical experience may have influenced outcomes, as well as each operator’s technical evolution over time.

Several clinically relevant variables were unavailable or incompletely recorded, which are priority parameters for future prospective multi-centre studies. Moreover, there was bias arising from case exclusion secondary to missing covariables. We lacked systematic data on flap weight, number and calibre of perforators, recipient vessels, and intraoperative adjuncts such as perfusion assessment, as well as mastectomy technique. Prior abdominal surgery was coded as a binary variable without scar pattern or location. Radiotherapy denoted prior chest-wall irradiation; timing relative to flap transfer, fields and dose, and whether the flap itself was irradiated postoperatively, were not consistently available. Chemotherapy timing was not uniform. Re-operation was defined as any unplanned return to the operating room at the treating surgeon’s discretion; indications and interventional radiology events were variably documented.

Statistical analyses were limited by low event counts. We used univariable models and complete-case analyses without multiplicity adjustment, so results are exploratory and at risk of residual confounding, type I error and, for rarer outcomes such as flap loss, type II error.

External validity was constrained. Data reflected DIEP activity at one site per country and did not capture surgeon activity at other institutions. Conversions to other reconstructive methods were excluded and only unilateral reconstructions were analysed, limiting generalisability to bilateral cases and other flap types.

## Conclusions

In this international, two-centre retrospective comparative cohort of unilateral, DIEP flap-only reconstructions, we observed between-centre differences in case-mix, workflow and outcomes. Operative time was longer in the United Kingdom and hospital stay was shorter, in a setting where enhanced recovery after surgery was in place throughout the study period. Immediate reconstruction was far more common in the United Kingdom, while the Netherlands centre used a co-surgeon model and lean pathways. These system and workflow differences may contribute to observed variations, but causation cannot be inferred.

Across centres, longer operative time was associated with selected early and late minor complications, with small per-minute effects that become clinically meaningful when expressed per hour. Prior abdominal surgery was associated with late re-operation in the Netherlands cohort but not in pooled analyses. Prior radiotherapy was associated with late minor complications and fat necrosis in pooled and United Kingdom analyses, while major complication rates and flap loss did not differ by these factors. Breast-skin necrosis occurred predominantly as an early event after immediate reconstruction.

Taken together, these findings are hypothesis-generating and support pragmatic service priorities: careful preoperative planning in the presence of prior abdominal surgery or radiotherapy, attention to operative efficiency through team configuration (including co-surgeon operating) and process optimisation (including lean strategies), and perioperative pathways such as enhanced recovery after surgery. Future prospective studies with standardised capture of mastectomy technique, radiotherapy timing, operative adjuncts and re-operation indications, and with adequate power for uncommon outcomes, are needed to validate these observations and inform practice.

## Funding

No funding received.

## Ethical approval

All procedures performed in the study involving human participants were in accordance with the 1964 Helsinki declaration and its later amendments. This proposal has been presented to the local ethical committee (LTc) of the hospital Ziekenhuisgroep Twente (Almelo), which has granted a non-WMO license under registration number “ZGT24-17 DIEP”. This proposal was registered as an audit with anonymisation of data with the Royal Free London NHS Foundation Trust, United Kingdom.

## Declaration of competing interest

The authors declare that they have no conflict of interest.

## Appendix Supplementary materials

Image, application 1
