# Supplementary material for: Optimising the unilateral DIEP flap breast reconstruction: A United Kingdom-Netherlands multi-centre comparative study
Source: JPRAS Open. 2026 Jan 15;48:746–58. doi: 10.1016/j.jpra.2026.01.014 (PMC12891788; doi:10.1016/j.jpra.2026.01.014)
Supplement: Supplementary file 1 [file mmc1.pdf]

Supplements for manuscript entitled "***Optimising the unilateral DIEP flap breast reconstruction: A United Kingdom-Netherlands multi-centre comparative study***" are listed in consecutive order. Please find each supplement legend under its corresponding table.

Authors: Maximilian Jacobi, Petko Shtarbanov, Punn Tannirandorn, Zahra Ahmed, Stephen Hamilton, Shadi Ghali, Afshin Mosahebi, Sophie Riesmeijer, Dariush Nikkhah, Hinne Rakhorst

Supplement 1. A presentation of practical examples of how the NL centre has implemented lean process optimisation and the co-surgeon model from the start of the study period.

Phase	LEAN Principle Applied	Specific Workflow Action/Standard Work	Task Allocation/Personnel (Co-Surgeon Model)	Value Added/Mechanism of Efficiency
**Intra-operative (Setup/Preparation)**	Parallelisation	Flap harvest and recipient site prep in parallel: Second surgeon prepares the thoracic field while the fixed surgeon raises the flap.	First senior surgeon + Intern (Flap); Second Senior Surgeon(Recipient Site)	Eliminates sequential waiting; maximises surgeon utilisation.
**Intra-operative (Microsurgery)**	Standardisation/ Elimination of Motion	Joint anastomosis under the microscope with a preset layout; routine venous couplers; "redo-if-any-doubt" rule.	Both Senior Surgeons(Working together)	Maximises speed and quality (eliminates motion/search waste); ensures quality control and reduces risk of defect.
**Intra-operative (Dissection/Exposure)**	Decision Algorithms	IMA exposure: Split PM over widest intercostal space; rib rim removed only if corridor or vein calibre is inadequate.	First/Second Senior Surgeon(Applying pre-defined rules)	Prevents Over-processing; ensures standard work.
**Intra-operative (Closure)**	Task Standardisation/ Parallelisation	Closure divided: Senior surgeon closes deep fascia; interns perform subcuticular/skin closure of the abdomen.	One Senior Surgeon (Fascia); Interns (Skin/Subcuticular)	Reduces surgeon hand-offs (transport/waiting waste); frees senior time; provides balanced workload/avoids fatigue.
**Pre-operative**	Standardisation	CTA-based perforator planning; 30-min patient education session.	Lead Surgeon/Nursing Staff	Reduces intra-op searching/decision time (cognitive waste); standardises information.
**Peri-operative**	Eliminate Non-Value Steps	Catheter removal in OR/PACU, early line/drain rationalisation, Day-0 mobilisation.	OR/PACU/Ward Staff (Standardised team protocol)	Reduces waiting time and non-critical care steps; standardises recovery protocol (ERAS overlap).
**Overall**	Workload Balancing	All time-intensive steps are shared to secure a sustainable workload.	Alternating Pairs of Senior Surgeons	Prevents fatigue; maintains surgical quality during high-volume periods.

Supplement 2. Retrospective data collected.

**Baseline patient characteristics**<br>Age, body mass index in kgm^{-2}, positive smoking status (defined as smoking within six weeks of reconstructive surgery), past abdominal surgery.
**Diagnosed comorbidities**<br>Hypertension, diabetes mellitus.
**Other cancer therapies**<br>Chemotherapy (neoadjuvant and adjuvant), radiotherapy.
**Operative characteristics**<br>Immediate/delayed reconstruction, total operative time (defined as the interval between initial incision to final closure), immediate additional procedures (axillary lymph node clearance, contralateral mastopexy and others), use of venous couplers.
**Postoperative clinical complication types**<br>Total/partial flap loss, surgical infection, fat necrosis, seroma, haematoma, wound dehiscence, skin necrosis, herniae and bulging, medical complications.
**Dates**<br>Operation date, discharge date, postoperative days of incidence of any complication type, date of unplanned reoperation due to a complication type.

Supplement 2 legend

Complication types were divided according to the Clavien-Dindo classification (depending on whether re-operation with general or local anaesthesia was required), as well as early (<30 days) and late (>30 days) onset.

Supplement 3. The ERAS protocol adopted by the UK centre from the start of the study period.

Day 0

- PCA available OR Oxycodone BD for 72 hours only
- Pain Buster (if DIEP)
- Regular PO/IV paracetamol QDS
- 1-Hourly observations and flap monitoring
- IV fluids and urinary catheter in situ
- Prophylactic tinzaparin if not bleeding post op

Day 1 postoperatively

- PCA discontinued OR Oxycodone continues
- Regular PO codeine / Dihydrocodeine and PRN oramorph (if PCA) or oxynorm if on Oxycodone
- Regular PO paracetamol QDS
- Pain Buster
- Regular laxatives started (Senna and lactulose)
- 1-hourly flap monitoring
- IV fluids and urinary catheter in situ
- Prophylactic tinzaparin
- Eat and Drink as normal
- Mobilise out of bed and into chair

Day 2

- Pain Buster
- Regular PO codeine / Dihydrocodeine and PRN oramorph (if PCA) or oxynorm if on Oxycodone
- Regular PO paracetamol QDS
- Regular laxatives (Senna and lactulose)
- Two-hourly flap monitoring
- Aim to discontinue IV fluid and trial without catheter
- Prophylactic tinzaparin
- Bra on

Day 3

- Pain Buster to be removed
- Oxycodone to be STOPPED
- Regular PO paracetamol QDS
- Regular PO codeine / Dihydrocodeine and PRN oramorph (if PCA) or oxynorm if on Oxycodone
- Regular laxatives (Senna and lactulose)
- Two-hourly flap monitoring
- Prophylactic tinzaparin
- Drains removal

Day 4 to discharge

- Regular PO paracetamol QDS
- PRN PO codeine/tramadol and oramorph
- Regular laxatives (Senna and lactulose)
- If bowels not open - consider glycerin suppository
- Four-hourly flap monitoring

Supplement 4. Identifying predictors for postoperative flap loss (partial or total) within 30 days.

Variable assessed	Unit UK	Unit NL	Pooled
Logistic regression **Unadjusted OR (95% CI),** ***P***			
Operative time, min	1.003 (0.990-1.015), *P*=0.693	1.000 (0.990-1.010), *P*=0.983	1.001 (0.993-1.009), *P*=0.826
Age, years	1.12 (0.97-1.29), *P*=0.125	1.003 (0.91-1.11), *P*=0.952	1.04 (0.96-1.13), *P*=0.308
BMI, kgm^{-2}	0.94 (0.72-1.23), *P*=0.649	0.93 (0.71-1.23), *P*=0.627	0.94 (0.77-1.14), *P*=0.506
Chi-square **n in group affected by complication/total in group (%),** ***P***			
Positive smoking status	N/A	N/A	N/A
Hypertension and diabetes mellitus	1/11 (9.1%), *P*=0.344	1/17 (5.9%), *P*=0.963	2/28 (7.1%), *P*=0.522
Prior abdominal surgery	3/42 (7.1%), *P*=0.155	1/9 (11.1%), *P*=0.575	4/51 (7.8%), *P*=0.285
Past radiotherapy	2/31 (6.5%), *P*=0.376	2/45 (4.4%), *P*=0.554	4/76 (5.3%), *P*=0.864
Chemotherapy	2/34 (5.9%), *P*=0.461	2/48 (4.2%), *P*=0.458	4/83 (4.8%), *P*=0.979
Immediate procedure	3/72 (4.2%), *P*=0.821	N/A	N/A
Axillary lymph node clearance	N/A	N/A	N/A
Any additional procedures	N/A	N/A	N/A

Supplement 4 legend

Abbreviations: BMI = body mass index; CI = confidence interval; N/A = not available (no complications of the given type occurred in this group); NL = The Netherlands; OR = odds ratio; UK = United Kingdom.
